# Left Lower Lobectomy for Lung Cancer Complicated by Unilateral Absence of the Left Pulmonary Artery: A Case Report

**DOI:** 10.70352/scrj.cr.25-0526

**Published:** 2025-10-29

**Authors:** Sakiko Sato, Keisuke Kobayashi, Naoki Kawakami, Kazunari Inoue, Masaharu Inagaki, Yuichi Ishikawa

**Affiliations:** 1 Department of Thoracic Surgery, Tsuchiura Kyodo General Hospital, Tsuchiura, Ibaraki, Japan; 2 Department of Respiratory Internal Medicine, Tsuchiura Kyodo General Hospital, Tsuchiura, Ibaraki, Japan; 3 Department of Pathology, Tsuchiura Kyodo General Hospital, Tsuchiura, Ibaraki, Japan

**Keywords:** unilateral absence of pulmonary artery (UAPA), lung cancer, collateral circulation, bronchopleural fistula

## Abstract

**INTRODUCTION:**

Unilateral absence of the pulmonary artery (UAPA) is a rare congenital vascular anomaly, often diagnosed in childhood but sometimes remaining asymptomatic until adulthood. Its coexistence with primary lung cancer is exceptionally uncommon. Surgical resection in such cases poses risks due to absence of the pulmonary artery and hypertrophied systemic vessels, which may lead to bleeding or ischemic complications.

**CASE PRESENTATION:**

A 69-year-old woman with a history of unilateral interstitial pneumonia was referred for evaluation of a growing nodule in the left lower lobe. She was asymptomatic, with no prior hemoptysis or infection. Imaging revealed left-sided UAPA with systemic collateral perfusion. Two 18F-fluorodeoxyglucose (FDG)-avid pulmonary nodules were detected, raising suspicion for stage IA3 (S9) and IA2 (S8) lung cancer. To avoid the high morbidity associated with pneumonectomy while achieving oncologic control, a left lower lobectomy was performed. Intraoperative findings included hypertrophied systemic vessels and absence of the pulmonary artery, which required conversion to thoracotomy due to bleeding from collateral vessels forming the fused fissure and anatomical complexity. The bronchial stump was reinforced with a free fat pad. Although the initial postoperative course was uneventful, bronchoscopy on POD 90 revealed a bronchopleural fistula. The patient was managed conservatively with close observation, during which bronchial epithelial perfusion gradually recovered, and she has remained clinically stable without additional intervention.

**CONCLUSIONS:**

Management of asymptomatic adult UAPA complicated by ipsilateral lung cancer requires careful, case-specific surgical planning. Lobectomy was selected to reduce pneumonectomy-related risks. Implementing preventive measures—such as a pedicled muscle flap or pericardial fat pad for bronchial stump coverage—is advisable to reduce ischemic risk in such cases.

## Abbreviations

BPF
: bronchopleural fistula
FDG
: 18F-fluorodeoxyglucose
FEV1
: forced expiratory volume in 1 second
ICG
: indocyanine green
S8
: segment 8
S9
: segment 9
UAPA
: unilateral absence of the pulmonary artery
VATS
: video-assisted thoracoscopic surgery

## INTRODUCTION

UAPA is a rare congenital cardiovascular anomaly with an estimated incidence of approximately 1 in 200000 individuals.^1)^ It is typically diagnosed in childhood due to respiratory symptoms or associated cardiovascular anomalies. However, asymptomatic cases may remain undetected until adulthood. In the absence of direct pulmonary arterial flow, the affected lung is perfused by a network of systemic collaterals—most commonly hypertrophied bronchial, intercostal, internal thoracic, and phrenic arteries.^2,3)^ These fragile and hypertrophic vessels significantly increase the risk of intraoperative hemorrhage during thoracic surgery. Furthermore, resection of a hypoperfused lung can lead to ischemic complications in the bronchi due to altered vascular dynamics.

We present a case of left lower lobe lung cancer in a patient with left pulmonary artery agenesis who underwent left lower lobectomy and subsequently developed a BPF, currently being managed conservatively.

## CASE PRESENTATION

A 69-year-old woman with a history of unilateral interstitial pneumonia and unrecognized UAPA was referred for evaluation of a growing 2-cm pulmonary nodule in the left lower lobe, suspicious for primary lung cancer. Her medical history included dyslipidemia, with no prior respiratory infections or episodes of hemoptysis. She had a 40-pack-year smoking history.

Initial laboratory tests were unremarkable. Electrocardiography showed a regular sinus rhythm at 61 bpm. Transthoracic echocardiography revealed preserved left ventricular systolic function (ejection fraction 72%), with no wall motion abnormalities, pulmonary hypertension, or congenital cardiac defects.

**Fig. 1:**
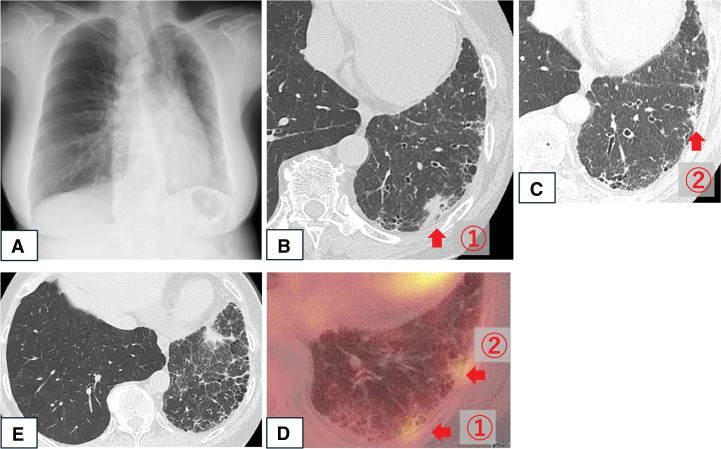
(**A**) Chest radiography showed left lung volume loss, leftward mediastinal shift, and reticular shadows. (**B**–**D**) CT showed ① a 25-mm solid nodule in left S9 with FDG uptake (SUVmax 4.02), ② a 10-mm nodule in left S8 with FDG uptake (SUVmax 3.03). (**E**) Unilateral interstitial pneumonia in the left lung. FDG, 18F-fluorodeoxyglucose; S8, segment 8; S9, segment 9

**Fig. 2:**
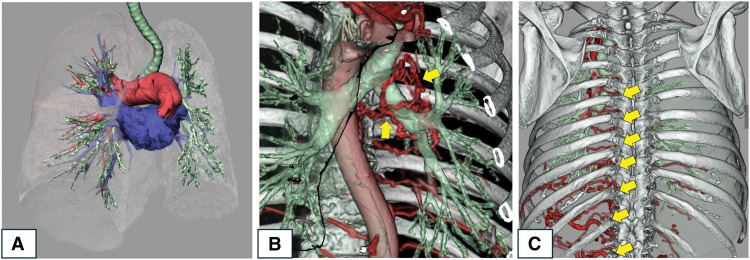
(**A**) Absence of the left main pulmonary artery. (**B, C**) Compensatory collateral perfusion via hypertrophied bronchial, intercostal, and diaphragmatic arteries on the left side (yellow arrow).

Pulmonary function testing showed a vital capacity of 2090 mL (96.3% predicted), a FEV1 of 1340 mL (82.2% predicted), and a FEV1/FVC ratio of 69.8%. Chest radiography showed signs of left lung volume loss, leftward mediastinal shift, and reticular/linear opacities suggestive of interstitial changes (**Fig. 1A**). CT identified a 25-mm solid nodule in S9 with high FDG uptake (SUVmax 4.02), and a 10-mm nodule in S8 with moderate uptake (SUVmax 3.03) (**Fig. 1B**–**1D**). Interstitial changes, including septal thickening, were confined to the left lung (**Fig. 1E**). CT also revealed congenital absence of the left main pulmonary artery, with compensatory perfusion via hypertrophied bronchial, intercostal, and diaphragmatic arteries (**Fig. 2A**–**2C**). Clinical staging was cT1cN0M0 (stage IA3) for the S9 lesion and cT1bN0M0 (stage IA2) for the S8 lesion, according to the 8th Edition of the TNM Classification of Malignant Tumors (UICC/AJCC).

Following multidisciplinary cancer board review, radiation therapy was deemed unsuitable due to underlying interstitial pneumonia. Given the patient’s asymptomatic status and absence of infection or hemoptysis, surgical resection was chosen. A left lower lobectomy was performed.

**Fig. 3:**
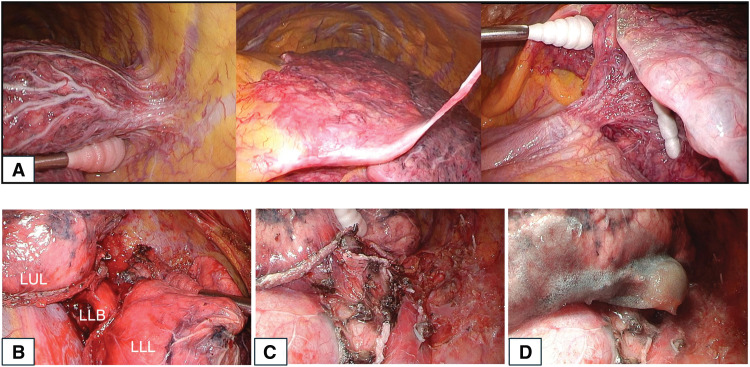
(**A**) Intraoperative findings of multiple dilated vessels on the visceral pleura, parenchymal interstitial changes, and prominent systemic collaterals. (**B**) Absence of a definite pulmonary artery on intraoperative inspection. (**C**) Completion of the left lower lobectomy. (**D**) Bronchial stump reinforcement using a free pericardial fat pad graft. LLB, left lower lobe bronchus; LLL, left lower lobe; LUL, left upper lobe

Intraoperatively, multiple dilated vessels were observed on the visceral pleura along with interstitial parenchymal changes and prominent systemic collaterals (**Fig. 3A**). These vessels were carefully ligated and divided using vessel-sealing devices and surgical clips. Frozen section analysis of a needle biopsy from the S9 lesion confirmed adenocarcinoma. The procedure was initiated as a multiportal VATS using a 5-cm access window and 2 additional ports, and was subsequently converted to a lateral thoracotomy with a 15-cm incision because the pulmonary artery was absent (**Fig. 3B**)—ordinarily a key anatomical landmark. In its absence, it was not possible to separate the interlobar fissure or safely encircle the lower lobe bronchus, and continued dissection only resulted in increased bleeding from collateral vessels forming the fused fissure. Subcarinal lymph node dissection was performed; however, dissection on the dorsal side of the carina was limited because of dense adhesions and collateral vessels, in order to avoid excessive bleeding from collateral circulation and to reduce the risk of bronchial ischemia. This allowed safer dissection and bronchial closure. The bronchial stump was reinforced with an autologous pericardial free fat pad following left lower lobectomy (**Fig. 3C** and **3D**). The operative time was 4 hours and 44 minutes, and the estimated intraoperative blood loss was 500 mL. The initial postoperative course was uneventful, and the patient was discharged on POD 9.

**Fig. 4:**
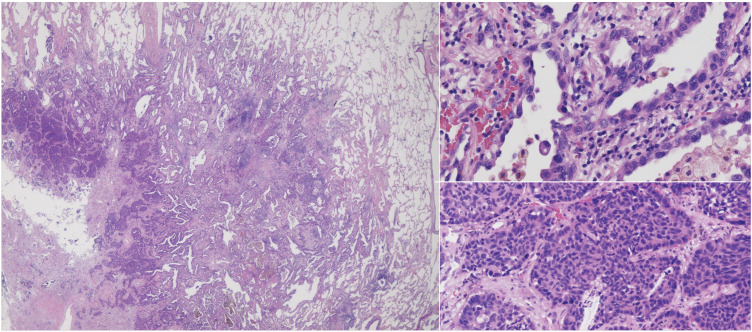
Double cancer arising from pulmonary fibrosis. Combined LCNEC and adenocarcinoma with giant cells in left S9 tumor (Lep:pap:LCNEC = 40:30:30%). Tumor size 27 × 16 mm = invasive size, pT2aN0M0 pl1v0ly1pm0 pStage IB. LCNEC, large cell neuroendocrine carcinoma; S9, segment 9

Pathological examination confirmed the absence of a pulmonary artery. Numerous thick-walled, smooth muscle-rich vessels surrounding the bronchioles were identified, consistent with bronchial arteries. Histopathology revealed that the S9 tumor was combined large cell neuroendocrine carcinoma and adenocarcinoma with giant cell features on a background of usual interstitial pneumonia (**Fig. 4**). The S8 tumor was lepidic adenocarcinoma.

**Fig. 5:**
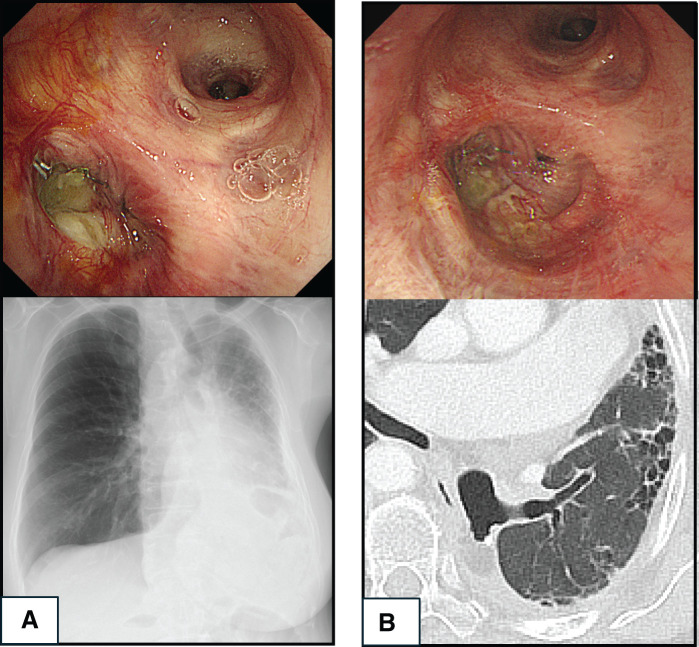
(**A**) Bronchoscopy on POD 90, revealing staple line dehiscence and fat pad exposure, consistent with BPF. Chest radiography showed no pneumothorax or air-fluid levels. (**B**) Bronchoscopy on POD 153, demonstrating epithelialization and fistula improvement. CT confirmed upper lobe re-expansion with no peristump air. Red dotted line indicates the fat pad overlying the bronchial stump. BPF, bronchopleural fistula

Routine bronchoscopy is usually performed about 1 week after anatomical lung resection, including lobectomy and segmentectomy, to evaluate the bronchial stump. In this case, surveillance bronchoscopy was continued because of the characteristic subepithelial vasculature observed preoperatively. On POD 90, outpatient bronchoscopy revealed staple-line dehiscence with exposure of the pericardial fat pad, consistent with a BPF (**Fig. 5A**). The patient remained afebrile and asymptomatic. Chest radiography showed no pneumothorax or air–fluid levels. As there was no cavity into which a drain could be inserted and no space for other interventions, conservative management was therefore selected, and the patient was followed up with CT imaging and bronchoscopy. By POD 153, bronchoscopy showed epithelialization and improvement of the fistula, with CT confirming upper lobe re-expansion and no evidence of peristump air (**Fig. 5B**). Ten months after surgery, the patient remains clinically stable with no evidence of recurrence.

## DISCUSSION

### Surgical approach in lung cancer with adult UAPA

The role of pneumonectomy in asymptomatic adult patients with UAPA remains uncertain. Specifically, for lung cancer occurring with UAPA, only 12 cases have been reported to date (**Table 1**).^4–15)^ Case series and systematic reviews suggest that pneumonectomy should be considered only for patients with significant symptoms such as hemoptysis, recurrent infections, or pulmonary hypertension. In a systematic review by Mattioni et al.,^16)^ postoperative complications occurred in approximately 36% of adult pneumonectomy cases, with 12% classified as Clavien–Dindo Grade IIIb, including reoperations for BPF and hemothorax—primarily in patients over 40 years old. In the context of lung cancer surgery, lobectomy is recommended for tumors ≤3 cm without nodal involvement as it is associated with lower morbidity and mortality compared with pneumonectomy.^17,18)^ Accordingly, in asymptomatic individuals with UAPA, lobectomy may be a more appropriate option for ipsilateral lung cancer, particularly when performed to minimize the bleeding risk associated with pneumonectomy.^10)^

**Table 1:** Summary of reported cases of lung cancer with unilateral absence of the pulmonary artery

Author (Year)	Age/Sex	Symptoms	UAPA relation	Side of UAPA	Tumor location	Treatment	Pathology	Postoperative complications
Mancebo and Wanner (1975)^4)^	49/F	Not described	Ipsilateral	Right	RUL	Not mentioned	Undifferentiated carcinoma	Not mentioned
Roman and Jones (1995)^5)^	54/M	Fever, chills, dyspnea, pleuritic chest pain, productive cough	Ipsilateral	Left	LLL	Pneumonectomy	Poorly differentiated adenocarcinoma	None
Anstadt et al. (2011)^6)^	67/F	Not described	Ipsilateral	Left	LUL	Lobectomy	Squamous cell carcinoma	None
Makdisi et al. (2015)^7)^	50/F	Recurrent hemoptysis	Ipsilateral	Right	RL (4 GGNs)	Pneumonectomy	Adenocarcinoma, pT1bN0, pStage IA	None
Watanabe et al. (2015)^8)^	76/F	Frequent episodes of pneumonia in childhood	Ipsilateral	Right	RLL	Lobectomy	Not mentioned	Not mentioned
Agzarian et al. (2019)^9)^	47/F	Fever, productive cough, hemoptysis, exertional dyspnea, left chest pain	Ipsilateral	Left	LL	Exploratory thoracotomy, biopsies, BSC	Adenocarcinoma, pleural metastases, pStage IV	Expired
Matsumoto et al. (2020)^10)^	80s/M	Asymptomatic	Ipsilateral	Right	RLL	VATS lobectomy	Non-small-cell lung cancer	None
Kononets et al. (2022)^11)^	59/F	Asymptomatic	Ipsilateral	Left	LLL	NAC(PTX+CDDP) → VATS pneumonectomy	Adenocarcinoma, ypT1N2M0, ypStage IIIA	None
Ito et al. (2010)^12)^	57/M	Not described	Contralateral	Left	RML	Lobectomy via thoracotomy	Adenocarcinoma	None
Zhang et al. (2016)^13)^	60/F	Chronic recurrent bronchitis	Contralateral	Right	LLL	Lobectomy via thoracotomy	Adenocarcinoma	Hypoxia, bloody tracheal excretions Expired (POD2)
Kim et al. (2018)^14)^	56/M	Not described	Contralateral	Left	RLL	VATS → lobectomy via thoracotomy	Adenocarcinoma	Dyspnea, hypoxia, oxygen therapy
Liu and Zhang (2021)^15)^	60/F	Asymptomatic	Contralateral	Right	LLL	VATS anatomical partial lobectomy	Invasive adenocarcinoma	None
Present case	69/F	Asymptomatic	Ipsilateral	Left	LLL	VATS → lobectomy via thoracotomy	Combined LCNEC and adenocarcinoma, pStage IB	BPF conservatively managed

BPF, bronchopleural fistula; BSC, best supportive care; CDDP, carboplatin; F, female; GGNs, ground-glass nodules; LCNEC, large cell neuroendocrine carcinoma; LL, left lung; LLL, left lower lobe; LUL, left upper lobe; M, male; NAC, neoadjuvant chemotherapy; PTX, paclitaxel; RL, right lung; RLL, right lower lung; RML, right middle lung; RUL, right upper lung; UAPA, unilateral absence of pulmonary artery; VATS, video-assisted thoracoscopic surgery

In this case, a 69-year-old asymptomatic patient underwent left lower lobectomy to minimize the hemorrhagic risk associated with pneumonectomy, while achieving adequate oncologic control. Adhesiolysis involving systemic collaterals was intentionally restricted to the extent necessary for lobectomy. This strategy balanced surgical safety and therapeutic efficacy in a high-risk anatomical setting.

### Risk and management of bronchopleural fistula in patients with UAPA

In UAPA, the risk of BPF after pulmonary resection is heightened due to impaired perfusion of the bronchial stump. Without a pulmonary artery, blood flow to the bronchial structures relies primarily on systemic collaterals.^2)^ Disruption of these vessels during surgery can lead to ischemic complications such as BPF.

**Fig. 6:**
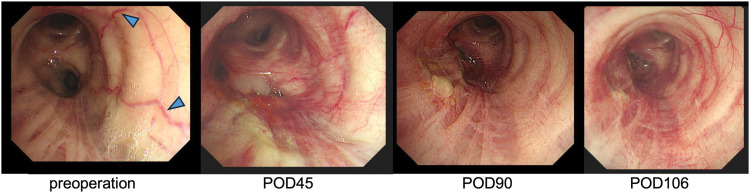
Time-course of bronchoscopic findings in the left main bronchus. Before surgery, prominent epithelial vascularization (blue arrow) is observed on the mediastinal side of the left main bronchus. By POD 45, the vessels become less distinct, disappear by 90 POD, and a bronchopleural fistula is observed. By POD 106, new capillary growth appears.

Time-course bronchoscopic observations revealed dynamic changes in the subepithelial vasculature of the left main bronchus. Preoperatively, prominent epithelial vessels were visible. By POD 45, these vessels became indistinct, and by POD 90—when the bronchial stump fistula was identified—they had disappeared. By POD 106, neovascularization in the form of capillaries was observed (**Fig. 6**). Notably, typical signs of ischemia reported in previous studies, such as whitish discoloration or black necrotic changes of the bronchial stump,^19)^ were not seen during the postoperative course. However, the transient disappearance of submucosal bronchial vessels followed by neovascularization, as visualized bronchoscopically, may indicate an episode of underlying ischemia that contributed to the development of the fistula in this case. Intraoperative perfusion assessment, such as ICG fluorescence, was not performed; however, limited subcarinal dissection was intended to preserve bronchial artery flow.^20)^ Such methods may provide useful objective information for evaluating the risk of postoperative ischemia in similar cases.

As the use of a free fat pad was followed by a bronchopleural fistula, alternative autologous tissue might have better preserved blood flow. In thoracoscopic lower lobectomy, we routinely cover the bronchial stump with a free pericardial fat pad due to its technical simplicity and adequate tissue retention, and the same strategy was applied in this case. Because subcarinal dissection was limited, we judged that the bronchial artery supply was preserved and therefore did not prepare an intercostal muscle flap. However, in retrospect, an intercostal muscle flap might have provided more reliable vascularized coverage. The literature suggests that a pedicled pericardial fat pad or an intercostal muscle flap—both of which are more resistant to ischemia—may have been more appropriate for bronchial stump coverage.^21)^ Additional preventive measures include minimizing the stump length.^22)^

## CONCLUSIONS

This rare case of lung cancer in a patient with UAPA illustrates the importance of individualized surgical planning. Lobectomy was chosen to balance oncologic curability with bleeding risk. Awareness of vascular anatomy and the application of a meticulous intraoperative technique—including limited adhesiolysis to minimize bleeding and the use of ischemia-resistant tissue for bronchial stump coverage—may help prevent postoperative complications such as BPF.

## DECLARATIONS

### Use of artificial intelligence (AI)

We used ChatGPT-4o (OpenAI) to assist with grammar and spelling correction, literature search, and for improving the clarity of English expressions throughout the manuscript. All AI-assisted content was carefully reviewed and edited by the authors. The authors take full responsibility for the accuracy and integrity of the final manuscript.

### Availability of data and materials

All data generated or analyzed during this study are included in this published article.
